# Hyperpolarized magnetic resonance shows that the anti‐ischemic drug meldonium leads to increased flux through pyruvate dehydrogenase in vivo resulting in improved post‐ischemic function in the diabetic heart

**DOI:** 10.1002/nbm.4471

**Published:** 2021-01-17

**Authors:** Dragana Savic, Vicky Ball, Lorenz Holzner, David Hauton, Kerstin N. Timm, M. Kate Curtis, Lisa C. Heather, Damian J. Tyler

**Affiliations:** ^1^ Cardiac Metabolism Research Group (CMRG), Department of Physiology, Anatomy and Genetics University of Oxford Oxford UK; ^2^ Oxford Centre for Clinical Magnetic Resonance Research (OCMR), Division of Cardiovascular Medicine, Radcliffe Department of Medicine University of Oxford Oxford UK; ^3^ Department of Physiology, Development and Neuroscience University of Cambridge Cambridge UK; ^4^ Metabolomics Research Group, Department of Chemistry University of Oxford Oxford UK

**Keywords:** cardiac function, diabetes, hyperpolarized MRI, langendorff perfusion, meldonium, metabolism, pyruvate dehydrogenase flux, streptozotocin

## Abstract

The diabetic heart has a decreased ability to metabolize glucose. The anti‐ischemic drug meldonium may provide a route to counteract this by reducing l‐carnitine levels, resulting in improved cardiac glucose utilization. Therefore, the aim of this study was to use the novel technique of hyperpolarized magnetic resonance to investigate the in vivo effects of treatment with meldonium on cardiac metabolism and function in control and diabetic rats. Thirty‐six male Wistar rats were injected either with vehicle, or with streptozotocin (55 mg/kg) to induce a model of type 1 diabetes. Daily treatment with either saline or meldonium (100 mg/kg/day) was undertaken for three weeks. in vivo cardiac function and metabolism were assessed with CINE MRI and hyperpolarized magnetic resonance respectively. Isolated perfused hearts were challenged with low‐flow ischemia/reperfusion to assess the impact of meldonium on post‐ischemic recovery. Meldonium had no significant effect on blood glucose concentrations or on baseline cardiac function. However, hyperpolarized magnetic resonance revealed that meldonium treatment elevated pyruvate dehydrogenase flux by 3.1‐fold and 1.2‐fold in diabetic and control animals, respectively, suggesting an increase in cardiac glucose oxidation. Hyperpolarized magnetic resonance further demonstrated that meldonium reduced the normalized acetylcarnitine signal by 2.1‐fold in both diabetic and control animals. The increase in pyruvate dehydrogenase flux in vivo was accompanied by an improvement in post‐ischemic function ex vivo, as meldonium elevated the rate pressure product by 1.3‐fold and 1.5‐fold in the control and diabetic animals, respectively. In conclusion, meldonium improves in vivo pyruvate dehydrogenase flux in the diabetic heart, contributing to improved cardiac recovery after ischemia.

## Graphical abstract

Glucose metabolism is decreased in the diabetic heart. Meldonium may provide a route to counteract this by reducing L‐carnitine levels and decreasing the uptake of fatty acids into the mitochondria, resulting in improved cardiac glucose utilization. Hyperpolarized magnetic resonance has revealed that meldonium treatment elevated pyruvate dehydrogenase flux in the diabetic heart, indicating an increase in cardiac glucose oxidation. In‐vivo glucose oxidation elevation was accompanied by an improvement in ex‐vivo post‐ischemic function, where meldonium elevated rate pressure following global ischemia.

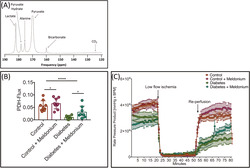

**Abbreviations**
3‐OHB: 3‐hydroxybutyrate
BPM: beats per minute
i.p.: intraperitoneal
LC‐MS: liquid chromatography‐mass spectrometry
NEFA: non‐esterified fatty acid
PDH: pyruvate dehydrogenase
RPP: rate pressure product
SD: standard deviation
STZ: streptozotocin
TAG: triglyceride
*T*_E_
: echo time
*T*_R_
: repetition time

## 1 INTRODUCTION

Diabetes has become increasingly prevalent in recent years, with a global incidence estimated to rise from 108 million people in 1980 to 700 million people by 2045.^1^ Of these people, it is suggested that 5‐15% will have type 1 diabetes.^2^ Diabetes is associated with an elevated risk of cardiovascular disease and heart failure,^3^, ^4^ and cardiovascular disease is the major cause of premature death in diabetic patients.^5^ Therefore, therapies that protect the heart in diabetes are urgently needed to reduce mortality.

Dysregulated cardiac substrate utilization and mitochondrial dysfunction are contributing factors to impaired cardiac function in the diabetic population.^6^, ^7^ In the healthy heart, 60‐70% of ATP production comes from fatty acids, and the remaining 30‐40% comes from glucose, lactate and amino acids. However, in diseases such as diabetes, this balance is shifted even further in favor of fatty acid oxidation.^8^, ^9^ Therefore, drugs that target this metabolic imbalance in the diabetic heart are of great therapeutic interest. One such drug is the anti‐ischemic agent meldonium (trimethylhydrazinium propionate), which is sold under the brand name Mildronate. The anti‐ischemic actions of meldonium are thought to originate from a switch in the balance of fuel utilization in the heart towards more oxygen‐efficient glucose metabolism. These effects arise from the impact of meldonium on the synthesis and transport of the amino acid derivative l‐carnitine.

l‐carnitine is required for the transport of long‐chain fatty acids into mitochondria via the carnitine shuttle. l‐carnitine accepts the fatty acid moiety from long‐chain acyl‐CoAs found in the cytoplasm, making them a suitable substrate for mitochondrial uptake via the carnitine‐acylcarnitine translocase within the inner mitochondrial membrane. Meldonium has been shown to reduce l‐carnitine availability both through inhibition of the l‐carnitine transporter OCTN2, inhibiting absorption and reabsorption of l‐carnitine in tissues, as well as through inhibition of the last step in the biosynthesis of l‐carnitine, γ‐butyrobetaine hydroxylase.

As such, treatment with meldonium can be hypothesized to reduce fatty acid uptake and oxidation in the mitochondrial matrix and provide a drive to increase glucose oxidation via the Randle cycle,^10^, ^11^ primarily through reduced acetyl‐CoA availability, which is known to inhibit glucose oxidation at the key regulatory step of pyruvate dehydrogenase (PDH).^12^ In a proof‐of‐concept study, we have shown previously that therapeutic strategies that improve mitochondrial pyruvate oxidation via increased PDH activity result in improved cardiac function in diabetes.^13^ However, clinically relevant compounds that improve cardiac PDH flux in diabetes are lacking. By modulating carnitine availability, meldonium may therefore provide a route to improve cardiac glucose utilization via increased PDH flux in diabetes.

This study aimed to investigate the effects of meldonium on in vivo cardiac metabolism and function in control and diabetic rodent hearts, using hyperpolarized MRS and CINE MRI. Hyperpolarized MRS is a novel technique, which can increase the in vivo sensitivity of MRS to detect ^13^C‐labeled metabolic substrates by more than 10 000‐fold.^14^ It therefore enables unprecedented real‐time visualization of the biochemical mechanisms of normal and abnormal metabolism, with measurement of instantaneous substrate uptake and enzymatic transformation in vivo.^15^ Following the injection of ^13^C pyruvate labeled at the first carbon position ([1‐^13^C]pyruvate), it is possible to see the metabolic production of carbon dioxide and bicarbonate (^13^CO_2_ and H^13^CO_3_^−^) through the PDH enzyme complex, yielding a marker of oxidation from carbohydrate sources. In addition, following injection of pyruvate labeled with ^13^C at the second carbon position ([2‐^13^C]pyruvate), it is possible to measure flux through the Krebs cycle, along with incorporation of acetyl‐CoA into acetylcarnitine, providing a marker of l‐carnitine availability. Hyperpolarized MRS therefore provides an ideal approach to explore the impact of meldonium treatment on the diabetic heart in vivo.

## 2 METHODS

### 2.1 Animal experimentation ethical approvals

All procedures (i) had local approval and (ii) conformed to the guidelines from Directive 2010/63/EU of the European Parliament on the protection of animals used for scientific purposes or the NIH Guide for the Care and Use of Laboratory Animals. Animal studies were conducted in accordance with the UK Animals (Scientific Procedures) Act (1986), PPL Number 30/3322, and local ethical guidelines (Medical Research Council Responsibility in the Use of Animals for Medical Research, July 1993).

### 2.2 Anesthetic agents

During all in vivo procedures, 2.5% isoflurane vol.:vol. in 1 L/min O_2_ was given via inhalation for anesthesia. An overdose of 5% isoflurane vol.:vol. in 2 L/min O_2_ was used for euthanasia; once the pedal and corneal reflexes ceased, the chest cavity was opened and the heart was excised.

### 2.3 Protocol

Thirty‐six healthy male Wistar rats (~200 g) were randomly divided into four groups. All animals were fasted overnight (food removed for 12‐15 h) and then either made diabetic with one intraperitoneal (i.p.) injection of streptozotocin (STZ, 55 mg/kg) or kept as controls via an injection of citrate buffer. Diabetes was confirmed by ensuring glucose levels higher than 110 mmol/L in urine samples using reagent test strips (Dirui, Jilin, China) for two consecutive days following STZ injection and by showing blood glucose levels higher than 8.3 mmol/L (Accu‐Chek Aviva, Roche, Basel, Switzerland) at 6 d after STZ injection. Two weeks after STZ/citrate buffer injection, all animals were initiated on daily morning i.p. treatment with either saline or meldonium (100 mg/kg/day). After two weeks of treatment, all animals were anesthetized with isoflurane and subjected to MRI and hyperpolarized MRS.

After three weeks of treatment, all animals were euthanized in the fed state with 5% isoflurane vol.:vol. in 2 L/min O_2_, followed by removal of the heart for Langendorff perfusion, with blood and tissue collected for analysis. Blood samples, taken from the chest cavity, were centrifuged (1200 g, 10 min, 4 °C) and plasma stored at −80 °C for later biochemical analysis. The right tibia length was measured and the kidneys and epididymal fat pads (from the posterior subcutaneous depots) were weighed.^16^ The hypertrophy index was calculated as the sum of the left and right kidney weights normalized to body weight. Other investigators have previously reported hypertrophy index in the literature when investigating STZ animals,^17^, ^18^, ^19^ as it is used as a progressive marker of diabetic renal disease.

### 2.4 CINE MRI

All rodents were imaged on a 7 T horizontal bore MRI instrument (Varian, Santa Clara, CA, USA), using a 72 mm ^1^H/^13^C volume transmit coil and a ^1^H four‐channel phased array surface receive coil (RAPID Biomedical, Rimpar, Germany). Eight to ten short‐axis (see Figure 2A later) slices (slice thickness, 1.6 mm; matrix size, 128 × 128; *T*_E_/*T*_R_, 4.6/1.45 ms; flip angle, 18**°**; number of averages, 4) were acquired with a CINE‐FLASH sequence.^20^ Left ventricular volumes at end systole and end diastole were derived using the free‐hand drawing function in ImageJ (NIH, Maryland, USA). For each heart, left ventricular mass, ejection fraction, stroke volume and cardiac output were calculated. The average myocardial mass of the left ventricle was obtained from the average of end diastolic and end systolic masses. Stroke volume was obtained from the difference between the end diastolic and end systolic volumes. All structural and functional parameters were also indexed to body weight to account for significant differences in body weight between the control and diabetic animals.

### 2.5 Hyperpolarized MRS

Experiments were performed between 7 am and 1 pm when rodents were in the fed state. Samples were prepared from 40 mg of either [1‐^13^C]pyruvic acid or [2‐^13^C]pyruvic acid (Sigma), doped with 15 mM trityl radical (OXO63, GE Healthcare, Chicago, USA) and 3 μl Dotarem (1:50 dilution, Guerbet, Villepinte, France) and hyperpolarized in a prototype polarizer (Oxford Instruments, Abingdon, UK) operating at a field strength of 3.35 T and a base temperature of 0.8‐1.2 K, with 30‐40 min of 100 mW microwave irradiation at approximately 94 GHz.^14^ The sample was subsequently dissolved in a pressurized and heated alkaline solution, containing 2.4 g/L sodium hydroxide and 100 mg/L EDTA (ethylenediaminetetraacetic acid) dipotassium salt (Sigma‐Aldrich), to yield a solution of 80 mM hyperpolarized sodium [1‐^13^C]pyruvate or [2‐^13^C]pyruvate with a polarization of 30% or 20% respectively, at physiological temperature and pH. From the resulting solution, 1 mL was injected over 10 s via a tail vein catheter into a rat located in the 7 T MRI system described above. Using the 72 mm ^1^H/^13^C volume transmit coil with a 40 mm two‐channel ^13^C surface receive array with an integrated preamp (RAPID Biomedical), cardiac ^13^C spectra were acquired using a simple ECG‐gated pulse‐acquire spectroscopy sequence over 60 s following the injection of the hyperpolarized pyruvate (repetition time, 1 s; excitation flip angle, 15**°**; sweep width, 13 021 Hz; acquired points, 2048; frequency centered on the C_1_ pyruvate resonance).^21^

Each rat received two injections (1 mL/injection), one with [1‐^13^C]pyruvate and one with [2‐^13^C]pyruvate, given in a random order and separated by at least 1 h. Following data acquisition, the ^13^C label from pyruvate and its metabolic products were summed over 30 s from the first appearance of pyruvate in the acquired spectra and fitted with the AMARES algorithm within jMRUI.^22^ Example summed spectra following injection of [1‐^13^C]pyruvate and [2‐^13^C]pyruvate are shown later in Figure 3A and 3B, with further example spectra from each of the groups studied shown in Supplementary Figures S1 and S2. Each of the metabolites was quantified as the ratio of the metabolites to either [1‐^13^C]pyruvate or [2‐^13^C]pyruvate.

PDH flux was calculated as the ratio of CO_2_ + bicarbonate to [1‐^13^C]pyruvate measured in the [1‐^13^C]pyruvate experiment, a measure that has previously been shown to correlate to PDH activity measured in an ex vivo enzymatic assay.^23^ Subsequently, to assess any changes within the Krebs cycle independent of changes in ^13^C flux through PDH (ie the ratio of CO_2_ + bicarbonate to [1‐^13^C]pyruvate), all metabolites obtained from [2‐^13^C]pyruvate were normalized to PDH flux measured in the [1‐^13^C]pyruvate experiment conducted in the same animal.

### 2.6 Langendorff perfusions

All animals were continued on the treatment protocol for one additional week following MRI/MRS, after which hearts were excised for a Langendorff ischemia/reperfusion protocol. The hearts were cannulated and perfused with warm oxygenated Krebs‐Henseleit buffer (37 °C) containing 11 mM glucose and 0.4 mM of palmitate, at a constant pressure of 100 mm Hg as described by Heather et al.^24^ A water‐filled PVC balloon, which was connected via a polythene tube to a calibrated pressure transducer and a PowerLab data acquisition system (AD Instruments, Oxford, Oxfordshire, UK), was inserted into the left ventricle to measure cardiac function. The balloon was inflated to an end diastolic pressure of 4‐8 mm Hg. Hearts were subjected to 20 min of normal flow (*t* = 1 min 20 s), followed by 30 min of a low‐flow ischemia (0.4 mL/min/gww, *t* = 21 min 50 s) and reperfused again at normal flow for another 30 min (*t* = 51 min 80 s). The hearts were freeze‐clamped with liquid‐nitrogen‐cooled Wallenberger tongs whilst still beating on the perfusion apparatus at *t* = 80 min. Cardiac function was assessed using the peak systolic pressure, end diastolic pressure, developed pressure (systolic minus end diastolic pressure), heart rate (beats per minute, BPM) and rate pressure product (RPP; developed pressure multiplied by heart rate).

### 2.7 Blood metabolites

Glucose concentrations were measured from fasted blood samples taken from the tail vein and acquired at one, two and five weeks after STZ injection. Non‐esterified fatty acids (NEFAs) were also measured in the fasted blood samples obtained five weeks after STZ/citrate buffer injection using an assay kit (Randox Laboratories, Crumlin, UK), respectively. Terminal fed blood samples were analyzed for 3‐hydroxybutyrate (3‐OHB), triglycerides (TAGs) and lactic acid using an ABX Pentra 400 (ABX Diagnostics, Kyoto, Japan).

### 2.8 Metabolomics

Terminal fed blood samples were assessed for low‐molecular‐weight metabolites with liquid chromatography‐mass spectrometry (LC‐MS) within the Department of Chemistry, University of Oxford. Plasma samples were filtered through molecular weight cut‐off filters (10 kDa) to remove proteins.^25^ The infranatant was recovered and evaporated to dryness under reduced pressure. Sample residue was then resuspended in acetonitrile:water (95%:5%). Authenticated standards for selected acylcarnitines (up to 1.0 μg/mL) were prepared using an identical method.

For LC‐MS, acylcarnitines were separated and resolved using hydrophobic‐interaction liquid chromatography‐mass spectrometry (HILIC). Samples were separated as previously outlined.^25^, ^26^ Briefly, samples were eluted using a binary solvent, acetonitrile:water (50%:50%) containing ammonium acetate (10 mM final concentration, Solvent A) and acetonitrile:water (95%:5%) containing ammonium acetate (10 mM final concentration, Solvent B). Samples were resolved using a linear gradient (10 min: 100% Solvent A to 100% Solvent B) and re‐equilibrated with 100% Solvent A. Putative compounds were identified with reference to authenticated standards for selected acylcarnitines using retention time, accurate mass and fragmentation pattern to identify individual compounds.^25^ Concentrations were calculated with reference to specific standard curves.

### 2.9 Statistics

All data are presented as mean ± standard deviation (SD) of the indicated number of rodents (*n*). Two‐way ANOVA was used for assessment of the effect of STZ injection and the effect of meldonium treatment. When an interaction term was significant in the two‐way ANOVA, post hoc multiple comparison testing using a Bonferroni correction was used to investigate the effect of meldonium treatment on both the control and diabetic groups. Differences between groups were considered statistically significant if *p* < 0.05.

## 3 RESULTS

### 3.1 Animal characterization

**FIGURE 1:**
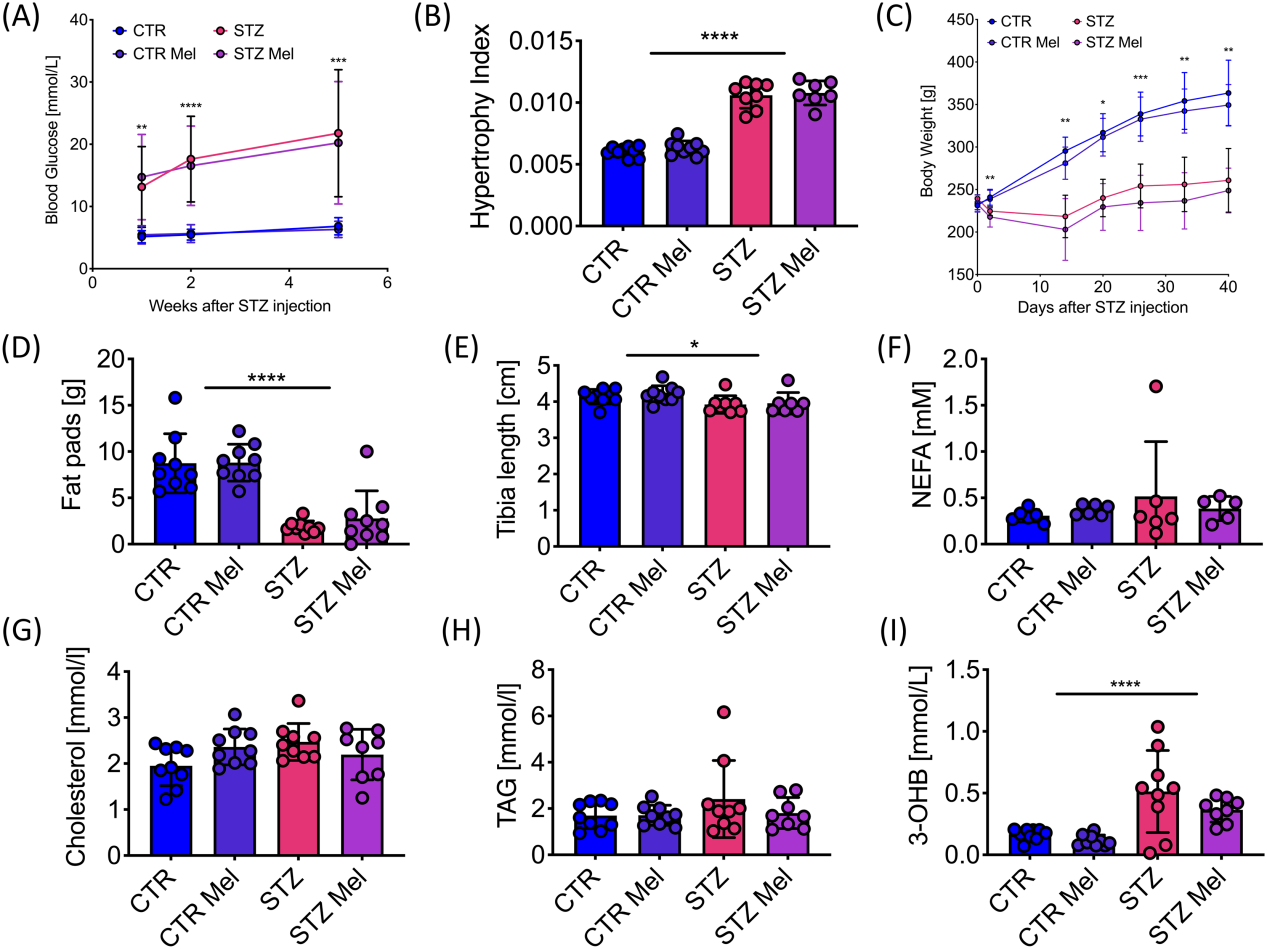
Animal characteristics five weeks after STZ‐induced diabetes (STZ) compared with citrate buffer injected controls (CTR); half of all animals were treated with meldonium (Mel) initiated at Week 2, generating four different groups. A, Fasted blood glucose concentration at 1, 2 and 5 weeks [mmol/L]. B, Hypertrophy index for kidneys; kidney weight normalized to body weight. C, Weight progression over time [g]. D, Epididymal fat pad weights at 5 weeks [g]. E, Tibia length at 5 weeks [cm]. F, Fasted NEFA levels at 5 weeks [mM]. G, Fed cholesterol levels at 5 weeks [mmol/L]. H, Fed TAG concentrations at 5 weeks [mmol/Ll]. I, Fed 3‐OHB concentrations at 5 weeks [mmol/l]. Data presented as mean ± SD. *p < 0.05, **p < 0.01, *** *p* < 0.001, ****p < 0.0001

STZ induction in male Wistar rats led to hyperglycemia (>13 mmol/L) observed one week after STZ injection, which gradually increased throughout the course of the experiment (Figure 1A). At five weeks after STZ injection, diabetic animals had markedly elevated glucose levels, which was associated with an elevated hypertrophy index of the kidneys (Figure 1B). Daily injections with meldonium had no significant impact on blood glucose levels or the kidney hypertrophy index.

Diabetic animals failed to gain weight over the course of the study, leading to a significant difference in body weight between controls and diabetics at all time‐points after the initial weight matching (Figure 1C). Lack of weight gain in the diabetic animals was attributed primarily to a reduction in fat mass as indicated by a 4.7‐fold reduction in epididymal fat pad weight (Figure 1D). There was also a small, but significant, reduction in lean mass as measured by a decreased tibia length at the terminal time‐point in the diabetic animals (Figure 1E). No differences in NEFA, TAG or cholesterol concentrations were observed in the diabetic animals; however, STZ injection resulted in a significant elevation in the levels of the ketone body, 3‐OHB (Figure 1F‐I). Meldonium treatment had no significant effect on any of the plasma metabolites assessed.

### 3.2 Cardiac function

**FIGURE 2:**
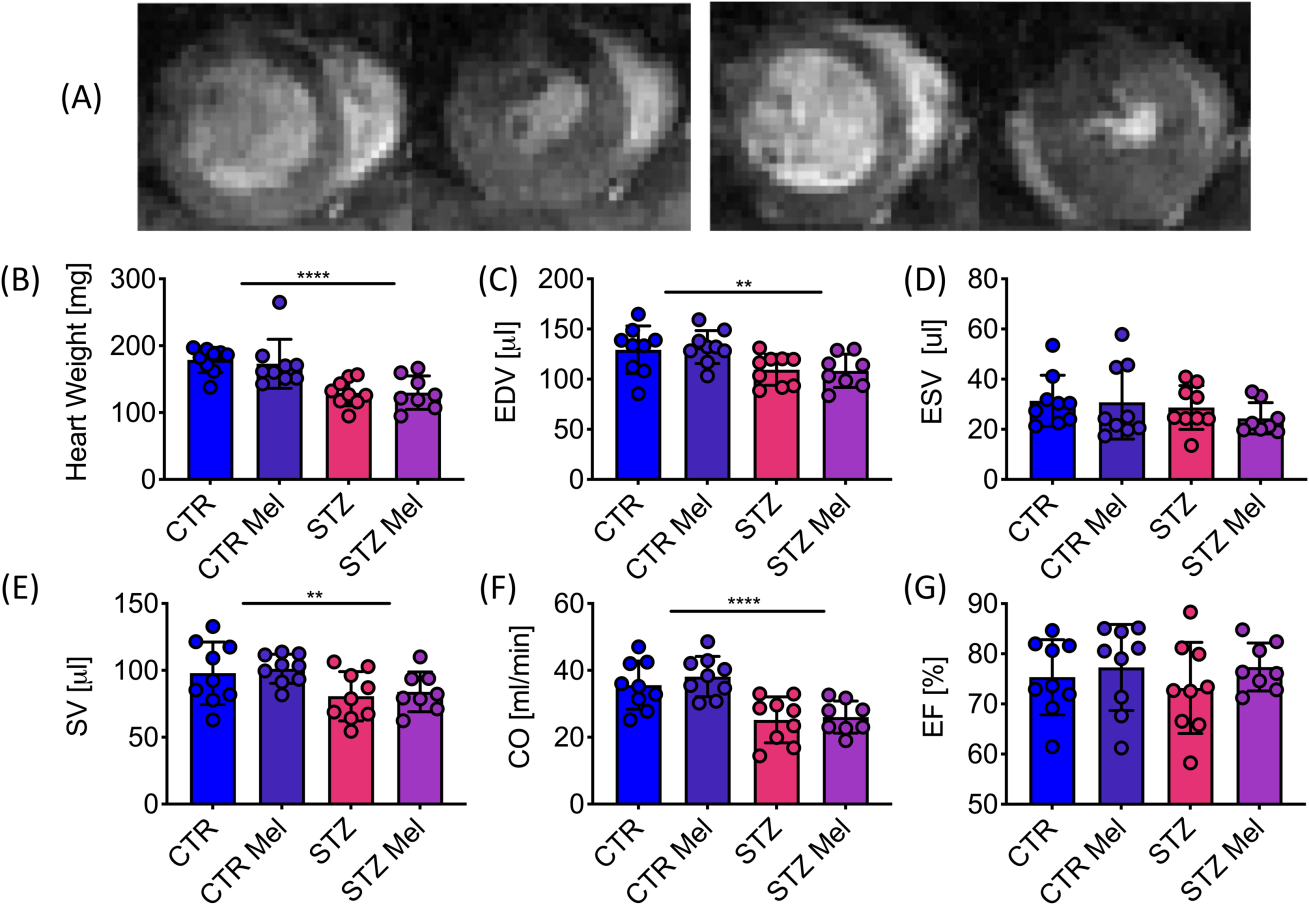
In vivo effects on cardiac function. A, Example CINE MRI images from diastole to systole, short‐axis view. B, Average myocardial wall mass [mg]. C, End diastolic volume (EDV) [μL]. D, End systolic volume (ESV) [μL]. E, Stroke volume (SV) [μL]. F, Cardiac output (CO) [mL/min]. (G) Ejection fraction (EF) [%]. Data presented as mean ± SD. **p < 0.01, ****p < 0.0001

Myocardial mass was reduced by 28% in the diabetic animals compared to the control animals (Figure 2B), but this was in proportion to the change in body weight, with no significant difference observed in the heart weight to body weight ratio (Table 1). End diastolic volume in the diabetic animals was reduced by 16%, leading to an 18% reduction in stroke volume and, when combined with a significantly decreased heart rate, a 29% lower cardiac output in comparison with the control animals (Figure 2C‐F). When differences in body weight were accounted for, this meant that a significant increase in stroke index was observed in the diabetic animals, which balanced the decrease in heart rate and led to no significant change in cardiac index (Table 1). Taken together with the lack of change in ejection fraction (Figure 2G), these structural and functional characterizations demonstrate that the diabetic hearts had no overt changes in systolic function apart from those induced by the reduction in body weight. Meldonium had no significant effect on any of the cardiac structural or functional parameters assessed.

**TABLE 1:** Cardiac function measured using CINE MRI. The table shows functional parameters, where most are normalized to body weight (BW). Data presented as mean ± SD. Two‐way ANOVA was undertaken and the effects of an interaction, STZ and meldonium are displayed with *p*‐values, where *p* < 0.05 was considered significant

	Control		STZ		STZ	Meldonium	Interaction
	Saline	Meldonium	Saline	Meldonium	*p*	*p*	*p*
HW/BW	0.56 ± 0.13	0.48 ± 0.07	0.54 ± 0.05	0.55 ± 0.06	0.46	0.17	0.12
Heart rate	370 ± 32	380 ± 32	310 ± 31	310 ± 32	**<0.0001**	0.59	0.70
End systolic volume/BW	0.098 ± 0.031	0.085 ± 0.037	0.12 ± 0.04	0.10 ± 0.022	0.072	0.18	0.82
End diastolic volume/BW	0.40 ± 0.09	0.37 ± 0.02	0.46 ± 0.05	0.46 ± 0.07	**0.0012**	0.42	0.35
Stroke volume/BW (stroke index)	0.31 ± 0.083	0.28 ± 0.022	0.34 ± 0.064	0.36 ± 0.06	**0.017**	0.95	0.30
Cardiac output/BW (cardiac index)	0.11 ± 0.028	0.11 ± 0.014	0.10 ± 0.019	0.11 ± 0.014	0.75	0.93	0.41

Abbreviation: HW, Heart weight.

### 3.3 Cardiac metabolism

**FIGURE 3:**
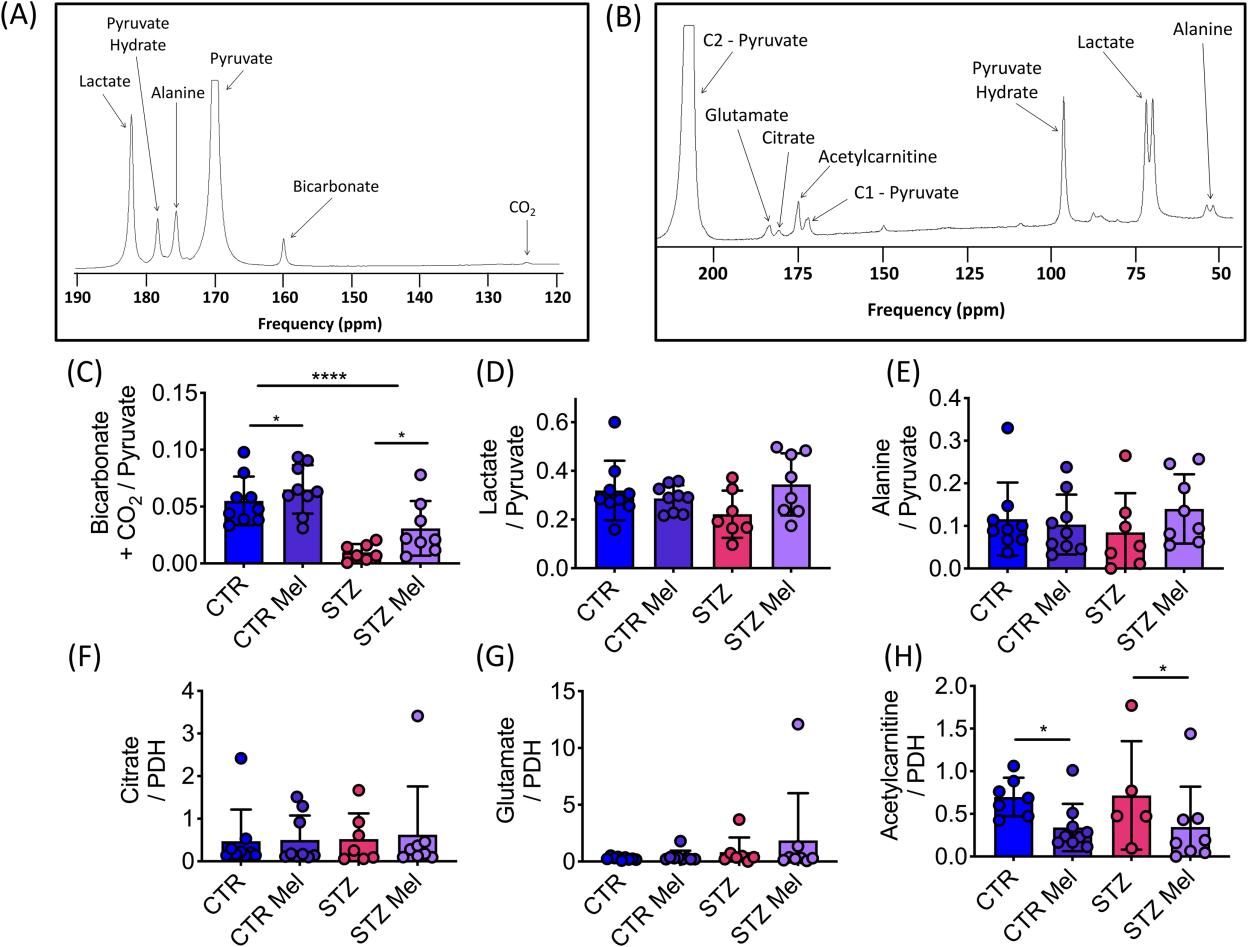
In vivo effects on cardiac metabolism. A, Example of a 30 s summed [1‐^13^C]pyruvate spectrum. B, Example of a 30 s summed [2‐^13^C]pyruvate spectrum. C, Bicarbonate + CO_2_/pyruvate ratio, a marker of PDH flux. D, Lactate/pyruvate ratio. E, Alanine/pyruvate ratio. F, Citrate/pyruvate ratio normalized to PDH‐flux. G, Glutamate/pyruvate ratio normalized to PDH‐flux. H, Acetylcarnitine/pyruvate ratio normalized to PDH‐flux. Data presented as mean ± SD. *p < 0.05, ****p < 0.0001

As has been previously observed,^27^ PDH flux, as assessed by bicarbonate and CO_2_ production from the injected hyperpolarized [1‐^13^C]pyruvate, was significantly reduced in the diabetic heart. In addition, meldonium treatment led to a significant increase in PDH flux, by approximately threefold in the diabetic animals and 1.2‐fold in the control animals (Figure 3C). Neither STZ injection nor meldonium treatment had any effect on the incorporation of the ^13^C label from [1‐^13^C]pyruvate into either lactate or alanine (Figure 3D and 3E).

After normalization for the differences observed in PDH flux, and thus transfer of the ^13^C label into the TCA cycle, no significant differences in ^13^C label incorporation into citrate or glutamate were observed in any of the groups (Figure 3F‐H). However, meldonium treatment led to a 2.1‐fold reduction in the incorporation of the ^13^C label into acetylcarnitine in both control and diabetic animals (Figure 3H), indicating a reduced availability of l‐carnitine in the meldonium treated heart.

### 3.4 Plasma metabolomics

**FIGURE 4:**
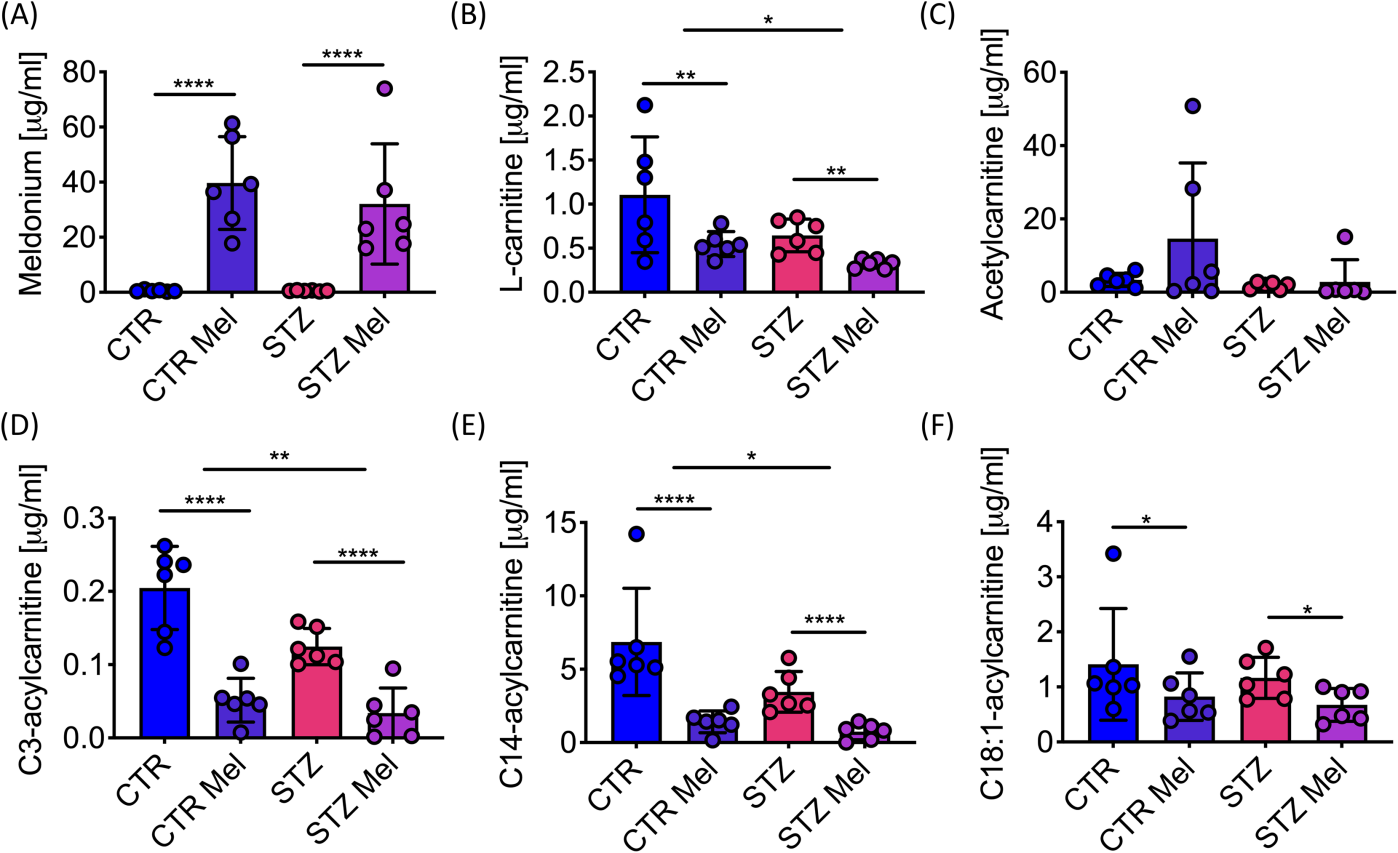
A, Meldonium concentration levels in plasma [μg/mL]. B, Free l‐carnitine concentration in plasma [μg/mL]. C, Acetylcarnitine concentration levels in plasma [μg/mL]. D, C3‐acylcarnitine (propionylcarnitine) concentration levels in plasma [μg/mL]. E, C14‐acylcarnitine (tetradecanoylcarnitine) concentration levels in plasma [μg/mL]. F, C18:1‐acylcarnitine (stearoylcarnitine) concentration levels in plasma [μg/mL]. Data presented as mean ± SD. *p < 0.05, **p < 0.01, ****p < 0.0001

As expected, meldonium treatment led to a significant elevation in the plasma levels of meldonium and a significant reduction in plasma l‐carnitine in both control and diabetic animals. This reduction was observed in addition to a significant reduction in l‐carnitine levels due to STZ‐induced diabetes (Figure 4A and 4B). Similar patterns to those seen in l‐carnitine levels were also observed with short‐chain (C3) and long‐chain (C14/C18) acylcarnitine species (Figure 4C‐F). Plasma levels of all acylcarnitine species assessed are presented in Table 2.

**TABLE 2:** Metabolomics were undertaken in the plasma of the animals. Different lengths of acylcarnitine species are displayed. Data presented as mean ± SD. Two‐way ANOVA undertaken and the effects of an interaction, STZ and meldonium are displayed with p‐values, where *p* < 0.05 was considered significant

	Control		STZ		STZ	Meldonium	Interaction
Metabolites [μg/ml]	Saline	Meldonium	Saline	Meldonium	*p*	*p*	*p*
Meldonium	0.54 ± 0.33	40 ± 0.17	0.55 ± 0.17	32 ± 22	0.51	**<0.0001**	0.51
l‐carnitine	1.11 ± 0.66	0.55 ± 0.14	0.65 ± 0.19	0.32 ± 0.05	**0.026**	**0.0059**	0.41
Acetylcarnitine	3.4 ± 1.8	15 ± 21	1.8 ± 0.090	2.8 ± 6.0	0.15	0.18	0.26
C3‐acylcarnitine	0.20 ± 0.057	0.052 ± 0.030	0.12 ± 0.025	0.034 ± 0.034	**0.0055**	**<0.0001**	0.061
C5‐acylcarnitine	0.11 ± 0.028	0.11 ± 0.014	0.10 ± 0.019	0.11 ± 0.014	0.29	0.78	0.33
C6‐acylcarnitine	12 ± 2.7	3.6 ± 1.8	5.2 ± 1.4	2.6 ± 2.1	**0.0002**	**<0.0001**	**0.0027**
C14‐acylcarnitine	6.9 ± 3.7	1.4 ± 0.74	3.5 ± 1.4	0.75 ± 0.55	**0.022**	**<0.0001**	0.11
C18‐acylcarnitine	2.8 ± 2.5	1.0 ± 0.45	1.7 ± 0.64	1.0 ± 0.75	0.32	**0.035**	0.35
C18:1‐acylcarnitine	1.4 ± 1.02	0.82 ± 0.43	1.7 ± 0.37	0.67 ± 0.30	0.43	**0.040**	0.85

### 3.5 Post‐ischemic recovery

**FIGURE 5:**
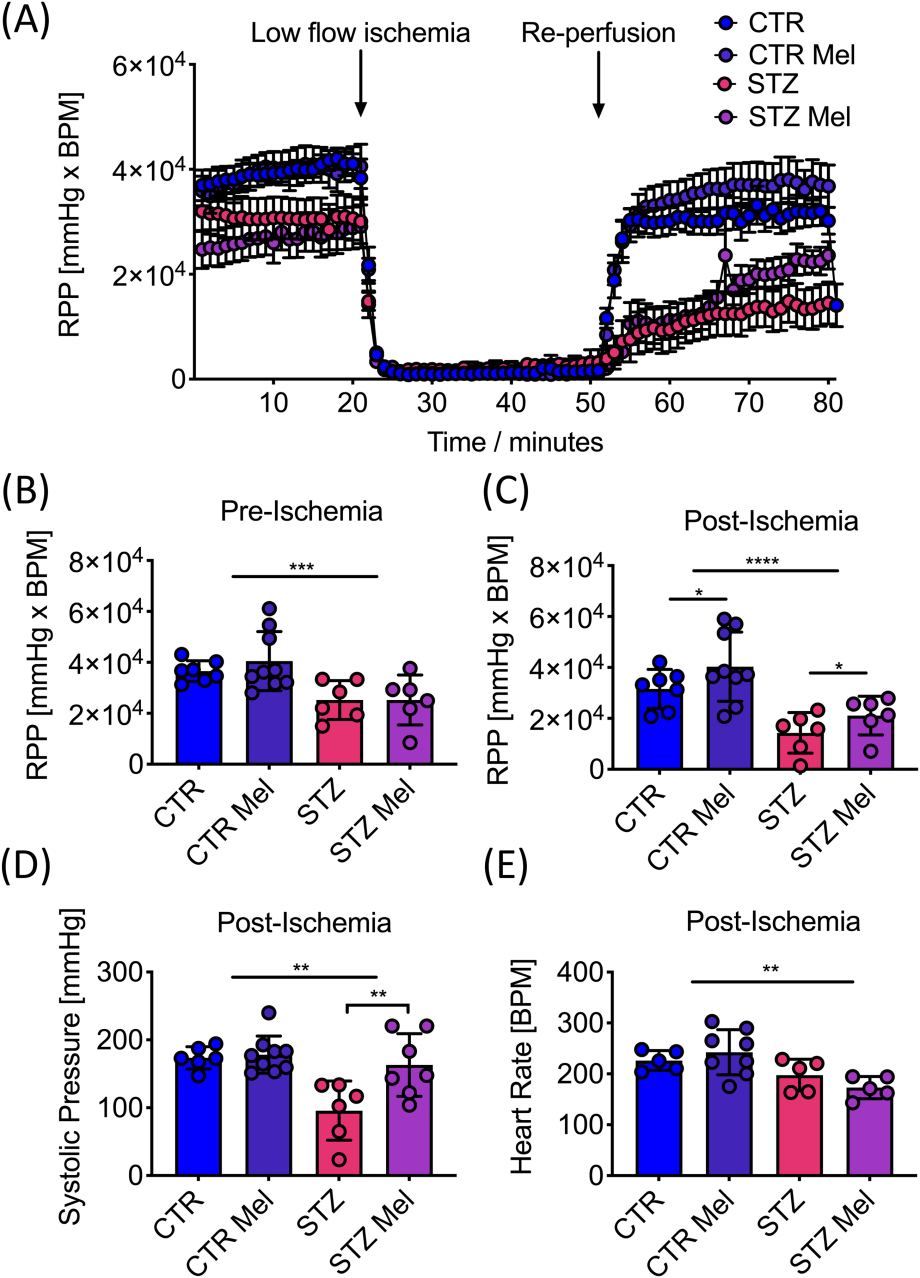
A, RPP over time [mm Hg × BPM]. Before ischemia (*t* = 1 min 20 s), low‐flow ischemia (*t* = 21 min 50 s), after ischemia (*t* = 52 min 80 s). B, RPP before ischemia [mm Hg × BPM]. C, RPP after ischemia [mm Hg × BPM]. D, Developed pressure after ischemia [mm Hg]. E, Heart rate after ischemia [BPM]. Data presented as mean ± SD. *p < 0.05, **p < 0.01, ***p < 0.001, ****p < 0.0001

As a marker of cardiac function, RPP was 31% lower in the diabetic animals before ischemia and this functional impairment worsened after ischemia with RPP 55% lower in the diabetic hearts (Figure 5A‐C). The observed impairment in post‐ischemic function in the diabetic heart was due to reductions in both systolic pressure (55% reduction) and heart rate (13% reduction) when compared with the control animals (Figure 5D and 5E).

However, meldonium treatment led to a significant elevation in post‐ischemic RPP by 1.3‐fold in the control animals and 1.5‐fold in the diabetic animals (Figure 5C). This improvement in post‐ischemic function was driven by small (but non‐significant) increases in both developed pressure and heart rate in the controls (Table 3), whilst it was driven by a significant 70% elevation in systolic pressure in the diabetic hearts (Figure 5D).

**TABLE 3:** A Langendorff ischemia/reperfusion protocol was undertaken. The functional parameters obtained before ischemia, during ischemia and after ischemia are displayed. Data presented as mean ± SD. Two‐way ANOVA was undertaken and the effects of an interaction, STZ and meldonium (Mel) are displayed with *p*‐values, where *p* < 0.05 was considered significant

	Control		STZ		STZ	Mel	Interaction
	Saline	Meldonium	Saline	Meldonium	p	p	p
*Before ischemia*							
RPP [mm Hg x BPM]	37 000 ± 4000	41 000 ± 11 000	25 000 ± 8000	25 000 ± 10 000	0.0008	0.57	0.57
HR [BPM]	256 ± 25	257 ± 25	221 ± 20	216 ± 31	0.003	0.84	0.75
Developed pressure [mm Hg]	147 ± 20	152 ± 40	112 ± 31	118 ± 48	0.027	0.69	0.94
Systolic pressure [mm Hg]	147 ± 19	151 ± 37	98 ± 60	118 ± 49	0.021	0.48	0.65
Diastolic pressure [mm Hg]	−2.0 ± 13	2.0 ± 7.0	−0.031 ± 5.7	−6.4 ± 19	0.49	0.80	0.27
*Ischemia*							
Systolic pressure [mm Hg]	28 ± 15	29 ± 20	55 ± 25	17 ± 12	0.0039	0.62	0.61
Diastolic pressure [mm Hg]	17 ± 6	14 ± 20	43 ± 32	36 ± 17	0.0046	0.50	0.78
*After ischemia*							
RPP [mm Hg x BPM]	32 000 ± 8000	40 000 ± 13 000	14 000 ± 8000	21 000 ± 7000	<0.0001	0.045	0.82
HR [BPM]	226 ± 19	242 ± 44	198 ± 31	173 ± 22	0.0026	0.77	0.17
Developed pressure [mm Hg]	144 ± 29	158 ± 41	72 ± 39	141 ± 61	0.017	0.025	0.13
Systolic pressure [mm Hg]	174 ± 16	178 ± 27	96 ± 44	163 ± 46	0.0021	0.013	0.029
Diastolic pressure [mm Hg]	25 ± 18	14 ± 27	36 ± 39	−6.4 ± 62	0.76	0.090	0.31

## 4 DISCUSSION

### 4.1 Overview

Diabetic patients are more likely to develop ischemic heart disease and suffer increased mortality and morbidity following a myocardial infarction.^28^ Whilst the pathophysiology of this increased burden of disease is complex and multi‐factorial, alterations in cardiac metabolism, with a shift towards reduced glucose metabolism, are considered to play a significant role. Therefore, therapeutic interventions that target elevation of glucose metabolism may offer some benefit to the diabetic population. As an example, meldonium has been shown to be an effective anti‐ischemic drug, which is proposed to switch the balance of fuel utilization away from fatty acid oxidation and towards glucose oxidation. Meldonium has been shown to work by lowering myocardial free l‐carnitine and long‐chain acylcarnitine by more than 60%. This is proposed to lead to a suppression of free fatty acid oxidation, which should lead to a reciprocal increase in glucose oxidation and may account for the observed myocardial protection during ischemia.^29^

### 4.2 Diabetes

This study has shown that, as expected, STZ‐induced type 1 diabetic rats had hyperglycemia accompanied by an elevated hypertrophy index in the kidneys. These are common features of STZ‐induced diabetes, with elevated glucose levels already known to be apparent after 2 d.^30^ The elevated hypertrophy index is mostly accounted for by increased fluid intake, as shown by Bauman,^31^ with an element of increased renal mass considered to account for the rest. Furthermore, the STZ‐injected animals had reduced body weight accompanied by both significantly smaller fat pads and reduced tibia length. Cardiac structure (left ventricle mass) and function (stroke volume, cardiac output) were reduced in the STZ‐injected animals relative to controls, but these changes were proportionate to the reduction in body weight. When indexed to body weight, a slight increase in stroke index was observed to balance the significant reduction in heart rate, allowing the diabetic animals to maintain a normal cardiac index. As the CINE MRI techniques used in this study only allow assessment of systolic function, these findings do not exclude the possibility that there may have been an element of diastolic dysfunction, as has previously been seen in diabetic animal models when assessed using echocardiography.^13^ in vivo metabolism, assessed with hyperpolarized magnetic resonance, showed reduced PDH flux in the STZ injected animals, which has been shown previously five days after STZ induction.^27^

### 4.3 Meldonium treatment in diabetic animals

Meldonium treatment had minimal effects on physical and plasma metabolite parameters assessed in the STZ‐injected animals (eg blood glucose, body weight, hypertrophy index etc). Previous studies have shown that meldonium can reduce blood glucose levels in both rodent models of diabetes (STZ injection) and insulin‐resistant models of diabetes (Zucker obese rats), as well as in humans with type 2 diabetes.^32^, ^33^, ^34^ However, treatment in those studies was continued for longer periods (4‐12 weeks) than in our current study (3 weeks) and so it is possible that a longer period of treatment might have revealed these beneficial effects.

However, we did observe that meldonium induced an increase in flux through PDH, as indicated by enhanced production of ^13^C bicarbonate and ^13^CO_2_ following the injection of hyperpolarized [1‐^13^C]pyruvate. In addition, plasma metabolomics and the use of hyperpolarized [2‐^13^C]pyruvate revealed a reduction in l‐carnitine availability, leading to reduced incorporation of the ^13^C label into acetylcarnitine. Taken together, this suggests an increase in glucose oxidation in the meldonium‐treated diabetic heart and a possible shift away from fatty acid oxidation. However, the exact effect of meldonium on fatty acid oxidation is unclear, with a previous study showing the potential for increased fatty acid oxidation in the mitochondria of meldonium‐treated hearts.^35^ Unfortunately, we were unable to directly assess fatty acid oxidation in vivo as hyperpolarized MRI is currently unable to probe the oxidation of long‐chain fatty acids, although development work has shown the ability of the technique to probe the oxidation of short‐chain fatty acids and ketone bodies.^36^, ^37^, ^38^, ^39^, ^40^, ^41^, ^42^ Fatty acid oxidation can be assessed in the ex vivo perfused heart using either radiolabeled or ^13^C‐labeled fatty acids. However, such experiments are less physiological than experiments performed in vivo, as the results can be affected by the concentrations of different substrates (eg glucose, fatty acids) contained within the perfusion buffer and the lack of circulating hormones (eg insulin) seen in vivo.

Improvements in post‐ischemic function were observed in the ex vivo perfused heart with a 50% increase in RPP following a 30 min period of low‐flow ischemia. This result agrees with the work of Vilskersts et al^43^ and Sesti et al,^44^ who have shown that meldonium treatment leads to a smaller infarct size after ischemia/reperfusion. Meldonium treatment has also previously been shown to improve diastolic heart function and increase left ventricular ejection fraction in patients with type 2 diabetes, with blood glucose levels also reduced in these patients.^45^ The mechanistic link between these improvements and meldonium's effect on l‐carnitine availability has been demonstrated by simultaneous treatment with meldonium and l‐carnitine, which prevents these beneficial effects.^46^

### 4.4 Meldonium treatment in control animals

Meldonium treatment induced very few effects in the healthy control animals but it increased PDH flux by 20%, likely indicating increased glucose oxidation. The mechanism for this increase would again appear to be mediated by a reduction in fatty acid oxidation due to the reduced availability of l‐carnitine for transport of fatty acids into the mitochondria. In support of this, hyperpolarized MRS also revealed a reduction in mitochondrial l‐carnitine availability in the control animals, as demonstrated by a reduction in the incorporation of [2‐^13^C]pyruvate into acetylcarnitine, a finding supported by significant reductions in several acylcarnitine species in the plasma, including free l‐carnitine. Meldonium showed no differences in ex vivo cardiac function in the control hearts prior to ischemia; however, after ischemia RPP was elevated, suggesting an improved recovery. Such a finding indicates that, even in the control heart, a switch towards increased flux through PDH is beneficial during reperfusion. A similar finding has previously been shown by Liu et al when using the PDH activator dichloroacetate.^47^

### 4.5 Study limitations

First, the in vivo metabolic data in this study were acquired using cardiac localization achieved through a surface receive coil placed directly over the heart. Previous imaging studies have demonstrated that the bicarbonate signal (and therefore all metabolic products downstream of PDH) only originates from the heart^48^; however, there is a possibility that the recorded lactate resonance may include some contamination from the nearby liver and/or lactate that has washed into the sensitive region of the coil in the blood.^49^ Whilst this means that there is no impact on the key findings in this paper (namely increased in PDH flux and reduced incorporation of PDH derived acetyl‐CoA into acetylcarnitine in the meldonium‐treated heart), to conclusively prove that meldonium does not have an impact on the incorporation of the ^13^C label from [1‐^13^C]pyruvate into [1‐^13^C]lactate would require imaging studies to better localize the derived signals to the myocardial tissue.

Second, the concentration of pyruvate injected (80 mM) is supraphysiological and so there is the potential that the pyruvate may have a physiological impact. However, the actual dose of pyruvate delivered is relatively small (80 μmol) and is rapidly distributed, diluted and metabolized. Previously published work has measured the circulating concentration of pyruvate following the hyperpolarized injection and shown it to peak at 250 μM 1 min after the injection. Such a concentration is only four times higher than the baseline plasma pyruvate concentration of 60 μM and is in the physiological range of pyruvate concentrations reached during exercise.^23^

Finally, we choose to undertake the [1‐^13^C]pyruvate and [2‐^13^C]pyruvate experiments separately with the two injections separated by at least 1 h. These experiments could have been performed together using either a co‐polarized mixture of the individually labeled pyruvic acid preparations or using dual‐labeled [1,2‐^13^C]pyruvic acid, which would have reduced the number of injections that needed to be carried out.^50^ However, with such approaches, the [5‐^13^C]glutamate and [1‐^13^C]acetylcarnitine resonances overlap to some extent with the [1‐^13^C]lactate and [1‐^13^C]pyruvate peaks, respectively, making them difficult to measure when *B*_0_ homogeneity is poor. We therefore chose to undertake these as separate experiments to improve the clarity of the acquired spectra.

## 5 CONCLUSION

Therapeutic interventions aimed at restoring the normal balance of glucose and fatty acid oxidation in the diabetic heart have been suggested to have potential in preventing the increased cardiovascular mortality and morbidity associated with the disease. In this study, we have used the novel imaging approach of hyperpolarized magnetic resonance to show that the anti‐ischemic agent meldonium leads to an increase in in vivo flux through PDH in both the healthy and diabetic rodent heart. This potentially results in a switch in fuel utilization towards more oxygen‐efficient glucose metabolism and may account for the improved recovery after ischemia. Given the recent demonstration of the ability of hyperpolarized magnetic resonance to study alterations in metabolism in the human heart,^51^, ^52^ there is clear potential for such studies to be translated into clinical trials assessing the potential for metabolic therapies in the diabetic heart.

## CONFLICT OF INTEREST

No competing interest declared.

## Supporting information


**Figure S1** Example spectra from each of the groups studied following the injection of hyperpolarized [1‐^13^C]pyruvate. Each spectra was obtained by summing the 30 individual spectra acquired following the first appearance of the hyperpolarized [1‐^13^C]pyruvate resonance.
**Figure S2** Example spectra from each of the groups studied following the injection of hyperpolarized [2‐^13^C]pyruvate. Each spectra was obtained by summing the 30 individual spectra acquired following the first appearance of the hyperpolarized [2‐^13^C]pyruvate resonance.

## Data Availability

The data that support the findings of this study are available on request from the corresponding author. The data are not publicly available due to privacy or ethical restrictions.
