# Supplementary material for: Hyperpolarized magnetic resonance shows that the anti‐ischemic drug meldonium leads to increased flux through pyruvate dehydrogenase in vivo resulting in improved post‐ischemic function in the diabetic heart
Source: NMR Biomed. 2021 Jan 17;34(4):e4471. doi: 10.1002/nbm.4471 (PMC8609426; doi:10.1002/nbm.4471)

**Supplementary Information**

**Supplementary Figure – S1** – Example spectra from each of the groups studied following the injection of hyperpolarized [1-^13^C]pyruvate. Each spectra was obtained by summing the 30 individual spectra acquired following the first appearance of the hyperpolarized [1-^13^C]pyruvate resonance.


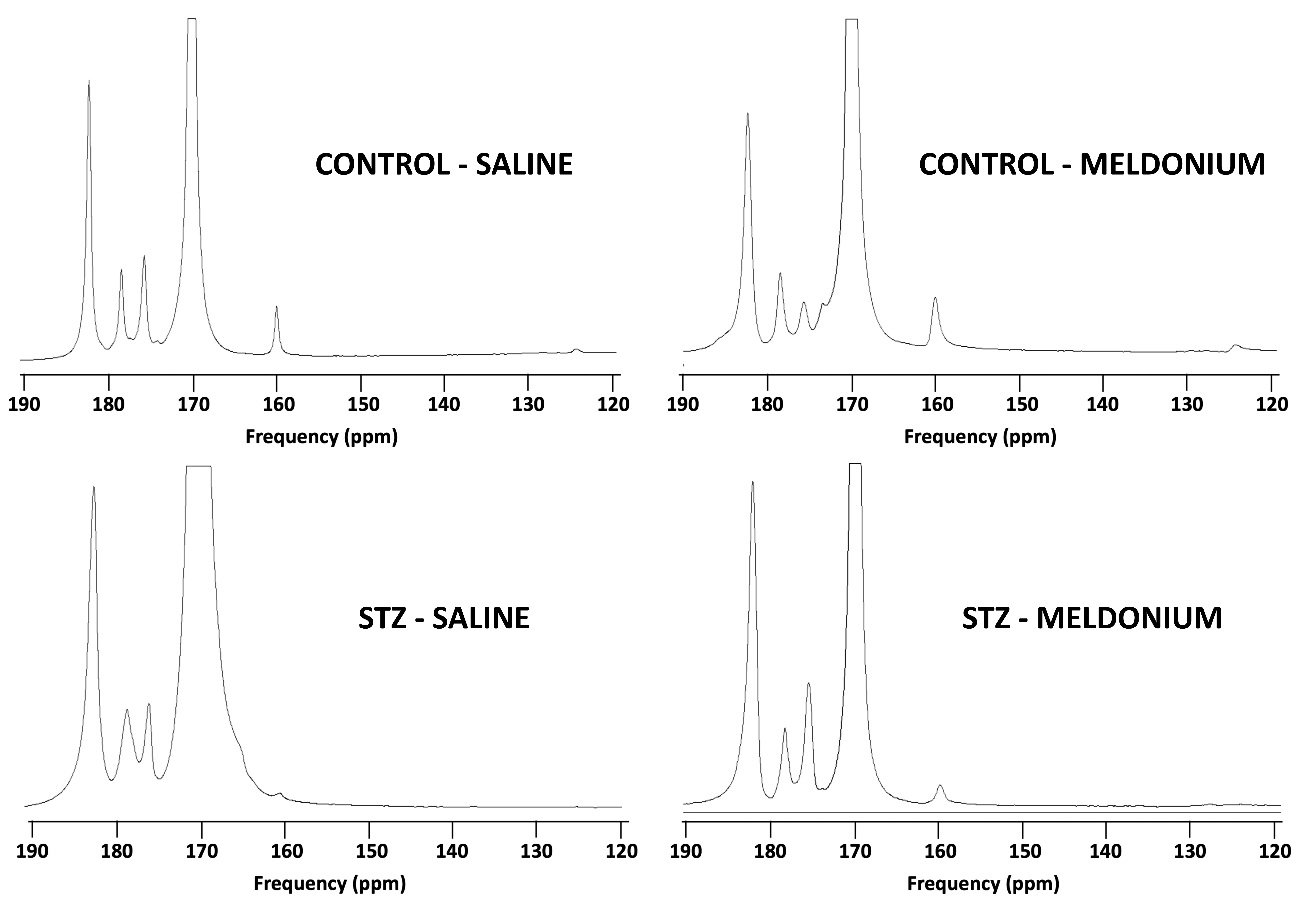


**Supplementary Figure – S2** – Example spectra from each of the groups studied following the injection of hyperpolarized [2-^13^C]pyruvate. Each spectra was obtained by summing the 30 individual spectra acquired following the first appearance of the hyperpolarized [2-^13^C]pyruvate resonance.


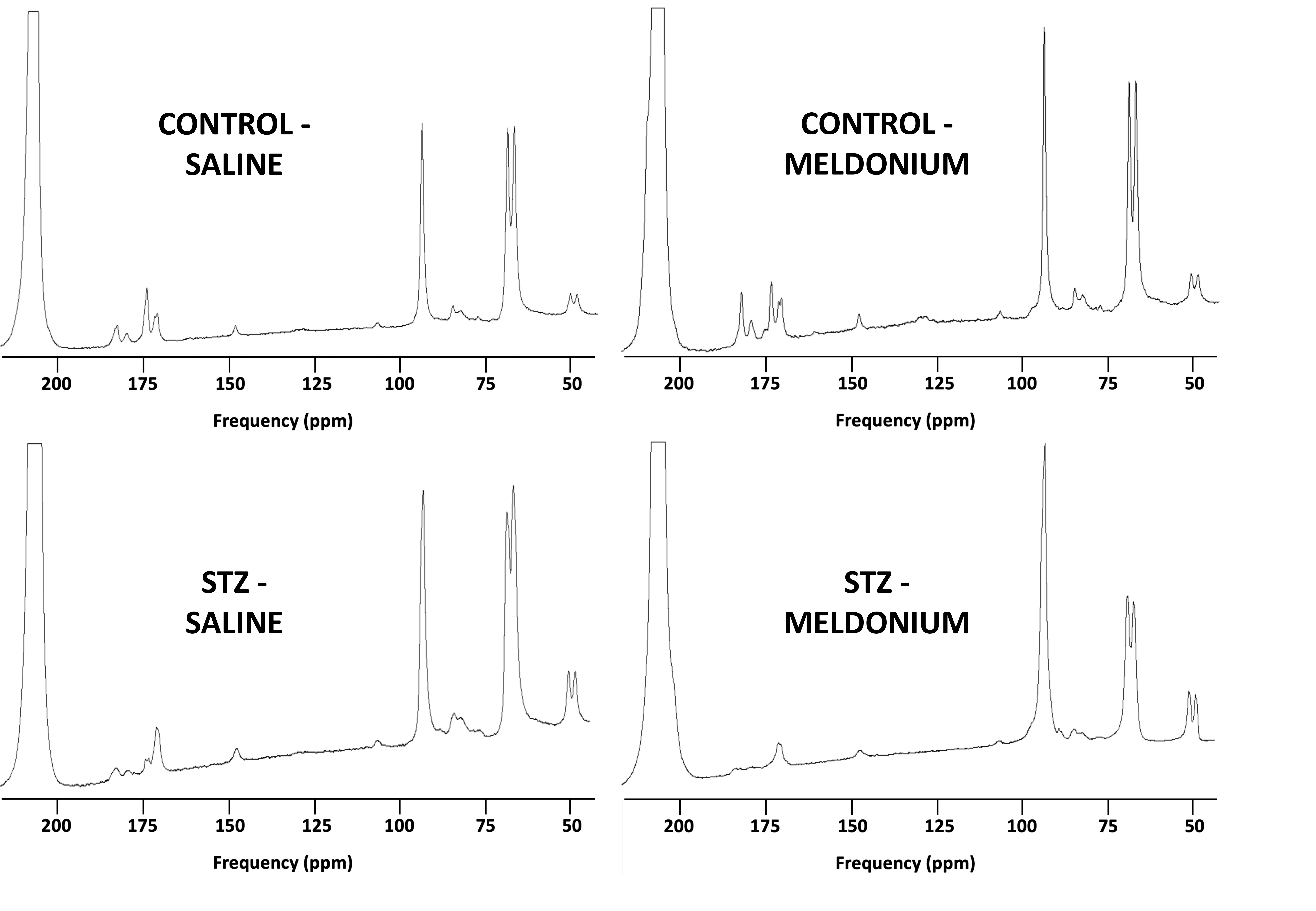

Supplement: Supplementary file 1 — Figure S1 Example spectra from each of the groups studied following the injection of hyperpolarized [1‐13C]pyruvate. Each spectra was obtained by summing the 30 individual spectra acquired following the first appearance of the hyperpolarized [1‐13C]pyruvate resonance. Figure S2 Example spectra from each of the groups studied following the injection of hyperpolarized [2‐13C]pyruvate. Each spectra was obtained by summing the 30 individual spectra acquired following the first appearance of the hyperpolarized [2‐13C]pyruvate resonance. [file NBM-34-e4471-s001.docx]
